# Cutaneous glomuvenous malformations in a family with dual *DICER1* and *GLMN* mutations: Case report and considerations

**DOI:** 10.1016/j.jdcr.2025.07.009

**Published:** 2025-08-05

**Authors:** Helen Z. Chen, Hafsa Zuberi, Julie Beasley, Cloyce L. Stetson, Brent Paulger

**Affiliations:** a Department of Dermatology, Texas Tech University Health Sciences Center, Lubbock, Texas; b School of Medicine, Texas Tech University Health Sciences Center, Lubbock, Texas; c Joe Arrington Cancer Research and Treatment Center, Lubbock, Texas; d Dermatology Associates of West Texas, Lubbock, Texas

**Keywords:** *DICER1* mutation, disease surveillance, *GLMN* mutation, glomuvenous malformations

## Introduction

*DICER1* syndrome, also known as pleuropulmonary blastoma familial tumor and dysplasia syndrome, is a rare autosomal dominant disorder (estimated prevalence of 1:10,600) caused by a mutation in the *DICER1* gene on chromosome 14q leading to downstream alteration of an RNase III endoribonuclease.^1^, ^2^, ^3^ Patients with this genetic mutation are at an increased risk for a range of tumors, particularly pleuropulmonary blastomas.^2^ Other systemic features of the gene mutation include development of Sertoli-Leydig cell tumors, cystic nephromas, pineoblastomas, multinodular goiters, and thyroid carcinomas.^3^^,^^4^ Management for patients with a *DICER1* mutation entails routine surveillance for tumor development including periodic thyroid ultrasounds, renal ultrasounds, tumor markers and chest x-rays, and genetic counseling.^2^^,^^3^

Cutaneous involvement is not currently recognized as part of the *DICER1* phenotype. However, dermatologists may encounter patients with *DICER1* mutations presenting with unrelated skin findings. We report a family with confirmed mutations in both *DICER1* and *GLMN*, another rare autosomal dominant condition. This co-occurrence raises the possibility of a shared or modifying genetic influence. While this could be coincidental, it may also suggest an under-recognized clinical overlap or interaction between these 2 gene mutations.

## Case report

**Fig 1:**
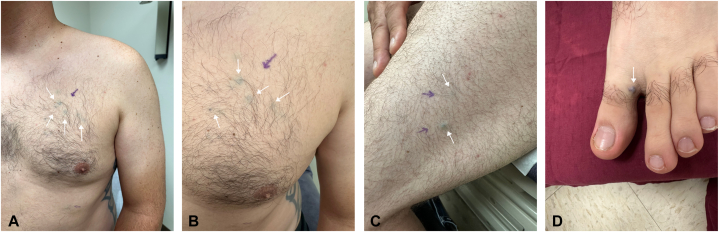
Clinical photos displaying *blue nodules* (demarcated with *white and purple arrows*) on our **(A** and **B)** patient’s chest, **(C)** lateral thigh, and **(D)** great toe.

**Fig 2:**
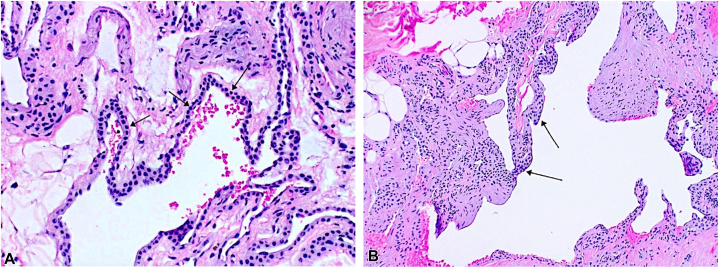
Hematoxylin and eosin (H&E) staining of the **(A)** left chest at 200× and **(B)** left abdomen at 80× magnification shows a venous malformation located in the deep dermis, composed of small, monomorphic cells without significant atypia that were glomus cells (*black arrows*). Mature vascular spaces are present surrounding the tumor cells, consistent with GVMs. *GVM*, Glomuvenous malformation.

**Fig 3:**
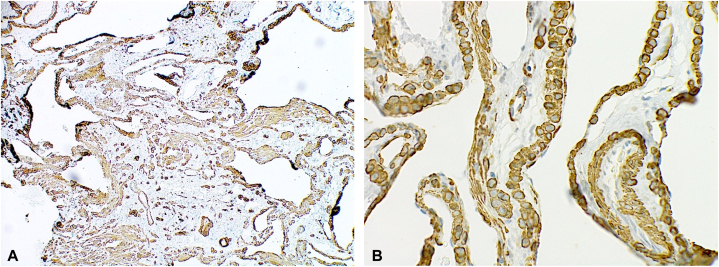
Smooth muscle actin staining that was strongly and diffusely positive throughout specimen at **(A)** 80× and **(B)** 400× magnification.

**Fig 4:**
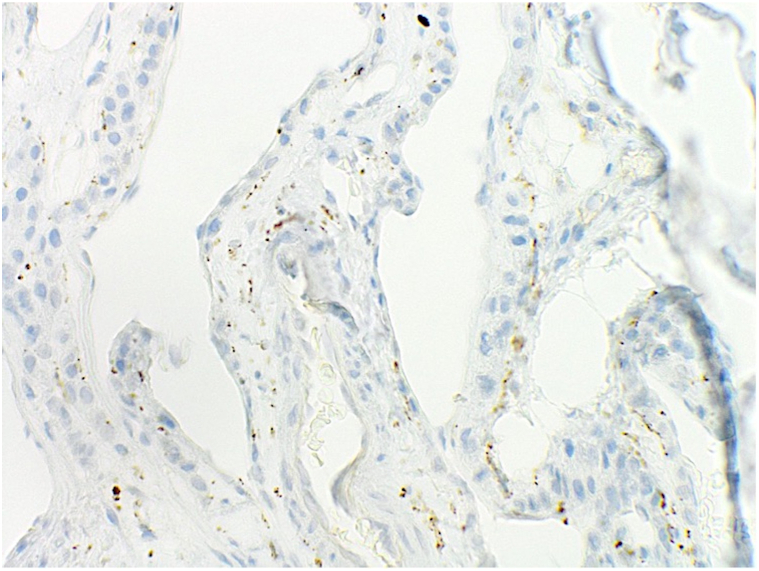
Synaptophysin staining which was negative at 400× magnification.

A 36-yearold Caucasian male with a history of a *DICER1* mutation (c.3583_3584delGA, p.D1195Lfs∗39) presented to our dermatology clinic for numerous asymptomatic blue nodules on the trunk and extremities, which have been present since his teenage years. Over the past year, he had noticed the development of additional nodules and was subsequently referred to dermatology. Physical examination revealed multiple slightly compressible blue papules to nodules on the left chest, left abdomen, left anterior hip, and upper and lower extremities bilaterally (Fig 1). The differential diagnosis included multiple hemangiomas, blue rubber bleb nevus syndrome, and other vascular neoplasms. Punch biopsies of lesions on the left chest and abdomen revealed tumors in the deep dermis composed of small, monomorphic cells without atypia and surrounded by mature vascular spaces (Fig 2). Smooth muscle actin immunohistochemical stain was strongly and diffusely positive while synaptophysin was negative, consistent with glomuvenous malformations (GVMs) (Figs 3 and 4).

At presentation, the patient had no systemic tumors commonly seen with *DICER1* syndrome, such as pulmonary or thyroid neoplasms. His family history was notable for 2 of his 3 children also testing positive for the *DICER1* mutation. One child with *DICER1* positivity had a history of cystic nephroma, and the other had a benign thyroid nodule. Interestingly, both *DICER1*-positive children reported similar blue nodules. Given the unusual presentation, additional genetic testing for *GLMN* and *TEK* mutations were pursued. The patient and his 2 children who were positive for the *DICER1* mutation were also found to carry a *GLMN* mutation, while testing negative for *TEK. TEK* mutations are associated with blue rubber bleb nevus syndrome, which can often present with multiple blue fluid-filled lesions that can affect the skin and gastrointestinal tract.

## Discussion

We describe a family with mutations in both *DICER1* and *GLMN*, presenting with multiple GVMs. While the cutaneous tumors may be explained by the *GLMN* mutation alone, the co-occurrence with *DICER1* raises questions about potential gene interaction, modifying effects, or shared pathogenic pathways. *GLMN*, or glomulin gene mutations, are typically associated with GVMs and are more readily identified in those lesions.^5^ In contrast, *GLMN* mutations are more difficult to detect in isolated glomus tumors.

GVMs typically present as painless blue-purple nodules, although they may become painful when compressed. In contrast, glomus tumors are characterized by paroxysmal pain, often triggered by temperature changes. Glomus tumors most commonly occur on the hands and feet where glomus bodies are most concentrated, whereas GVMs can appear across a broader anatomic distibution.^6^ GVMs usually present in young adults, with subungual tumor involvement more frequently seen in females and multiple lesions more common in males.^7^ Visceral organ involvement in patients with GVM or glomus tumors is rare, but when present, may affect the respiratory system, gastrointestinal tract, liver, pancreas, reproductive organs, and bones.^8^

Glomus tumors and GVM arising from soft tissue typically do not express synaptophysin, in contrast to glomus tumor originating in visceral organs such as the stomach, liver, esophagus, duodenum, and kidney.^8^^,^^9^ In documented pedigrees of familial glomus tumors, most have been linked to familial paragangliomas rather than the cutaneous glomangioma type. This distinction is critical, as the term “glomus tumor” is often used interchangeably for both conditions in the literature.^10^ The hereditary nature of paragangliomas is associated with mutations in succinate dehydrogenase, von-Hippel Lindau, or multiple endocrine neoplasia type 2, all of which were negative in our patient and family.

The presentation in this family prompts several considerations. First, the presence of both *DICER1* and *GLMN* mutations could be coincidental. Second, *DICER1* may influence the expressivity of *GLMN*-associated lesions, possibly altering their clinical presentation or severity. Third, there may be an unrecognized interaction between the 2 genes, either through a second-hit mechanism or modulation of tumor predisposition.

Our patient, despite carrying a pathogenic *DICER1* mutation, has not developed hallmark *DICER1*-associated tumors. Likewise, the affected children, although still young, have so far shown only limited systemic involvement. The daughter was diagnosed with a cystic nephroma, a classic early manifestation of *DICER1* syndrome, while the son has a TI-RADS 4 thyroid nodule, which was biopsied and found to be benign. Although thyroid nodules are common in the general population, they occur more frequently in children with *DICER1* mutation. Most *DICER1*-associated tumors occur in patients aged less than 40 years, with cystic nephromas typically seen in young children and adolescents, and pleuropulmonary blastomas primarily affecting infants and children under the age of 6.^1^ This raises the possibility that the *GLMN* mutation might modify the typical tumor spectrum or severity seen in *DICER1* syndrome. Alternatively, *DICER1* could enhance the skin findings seen with *GLMN* by contributing to the development of multiple GVMs.

Although speculative, these hypotheses underscore the need for further investigation into the potential interplay between these 2 rare mutations. Understanding such interactions may improve genetic counseling and surveillance strategies for patients with either mutation.

We recommend that clinicians managing patients with a known *DICER1* mutation consider including skin examinations in routine surveillance, particularly in those reporting blue nodules or other vascular-appearing lesions. If GVMs are identified, especially in atypical locations, additional genetic testing for *GLMN* may be warranted. Likewise, dermatologists encountering patients with multiple GVMs should obtain a thorough history and physical examination to assess for features suggestive of *DICER1*, which may prompt additional workup like imaging to evaluate systemic involvement and genetic counseling to assess familial risk.

## Conflicts of interest

None disclosed.
